# A Brief Modified Family-Based Treatment Intervention for Youth With Mild Eating Disorders: A Case Series

**DOI:** 10.3389/fpsyt.2020.00105

**Published:** 2020-03-05

**Authors:** Wendy Spettigue, Zizo Aldaqqaq, Leanna Isserlin, Brittany Bishop, Mark L. Norris, Darcie Valois, Nicole Obeid

**Affiliations:** ^1^ Department of Psychiatry, University of Ottawa, Ottawa, ON, Canada; ^2^ Faculty of Medicine, University of Ottawa, Ottawa, ON, Canada; ^3^ Children's Hospital of Eastern Ontario Research Institute, Ottawa, ON, Canada; ^4^ Department of Psychiatry, Queen's University, Kingston, ON, Canada; ^5^ Department of Pediatrics, University of Ottawa, Ottawa, ON, Canada

**Keywords:** eating disorders, adolescents, family based treatment, anorexia nervosa, bulimia nervosa, community treatment

## Abstract

**Background:**

Family-based treatment (FBT), an outpatient treatment which is typically offered for 6–12 months by specially trained therapists, is currently the first line treatment for adolescent anorexia nervosa and bulimia nervosa. The success of FBT for adolescents with moderate to severe eating disorders indicates a potential use for a short course of modified FBT which could be offered as an early intervention by nonspecialized community mental health counselors to adolescents with mild or subsyndromal eating disorders.

**Methods:**

In 2016, seven adolescents with mild eating disorders underwent a brief intervention in the form of five FBT-inspired therapy sessions (called ‘DREAMS' sessions). The DREAMS sessions consisted of five replicable family sessions given over 6 weeks, each with a specific area of focus for treatment, such as nutrition and eating disorder symptoms, mood, relationships and anxiety. Charts of these seven patients were reviewed in 2019 to determine whether this treatment might be worthy of further study.

**Results:**

Based on a review of the progress notes, all seven patients reported an improvement in intake, a decrease in ED symptoms and an improvement in mood by the end of the sessions. All seven families reported that the sessions had been beneficial.

**Conclusion:**

Early intervention is recommended for adolescents who present in the early stages of an eating disorder, yet there are no guidelines to recommend which treatment should be offered to this population. Further research is required to determine whether a short course of modified FBT, such as these five FBT-inspired ‘DREAMS' sessions, may be an effective intervention to offer to youth who present with mild eating disorders.

## Introduction

Eating disorders (EDs) are defined by the Diagnostic and Statistical Manual of Mental Disorders 5^th^ Edition (DSM-5) as life-threatening illnesses marked by morbid preoccupation with body weight and shape (1). The DSM-5 currently recognizes anorexia nervosa (AN), bulimia nervosa (BN), and other specified feeding or eating disorders as clinically significant and distinct EDs. In adolescent and young adult women, lifetime prevalence rates have been estimated at 0.3–0.9% for AN, 1–2% for BN, and up to 15% in total for all EDs (2, 3). Whether due to medical complications or the frequency of suicide, standardized mortality rates in AN are the highest of any psychiatric disorder and are 12 times higher than the annual death rate from all causes in females 15–24 years of age (4–6).

Studies highlighting the importance of early intervention for AN and other EDs demonstrate that prolonged periods of illness (along with low weight and poor psychosocial functioning) can raise mortality risk associated with AN (7). A paper by Le Grange and Loeb stresses the urgency of early identification and treatment of adolescent EDs in preventing disease progression (8). The article argues that adolescent subsyndromal EDs (those which do not meet DSM-5 diagnostic criteria) “are not only clinically significant in their present state, but may represent legitimate candidates for preventive efforts in light of: (i) a risk of progression from subthreshold anorexia nervosa (SAN) to AN or subthreshold bulimia nervosa (SBN) to BN; (ii) the detrimental effects on outcome of delaying treatment; and (iii) the refractory, severe nature of eating disorders once the diagnostic threshold is crossed….Given that AN is notoriously difficult to treat, and there are limited efficacy data for adolescent BN, attempts to disrupt these disorders in what is arguably their early phases is an important goal in preventing more chronic and treatment‐resistant forms of these disorders” (8). The authors provide a strong argument for the need for effective early intervention in adolescent EDs. Yet despite strong evidence for early intervention, there are no guidelines or evidence-based treatments for treating youth who present in the early stages (or subsyndromal stages) of an ED (9, 10).

Family-based treatment (FBT), as described by Lock et al. in *Treatment Manual for Adolescent AN: A Family Based Approach* (11), is well established as the gold standard for treatment of adolescent AN (9) and has even seen clinical success as a treatment for young adults with AN (12). FBT is an outpatient therapy consisting of three phases with the goal of restoring the health of adolescents through family support (11). Phase 1 focuses on empowering parents to take control of nutrition, while siblings provide support. In phase 2, the control over eating and physical activity is gradually shifted back to the adolescent. Phase 3 gives the therapist an opportunity to ensure that normal family life has resumed and to identify developmental challenges and associated coping mechanisms for the adolescent (11). Numerous studies have found FBT to be the most effective treatment for sustaining full remission of AN symptoms and generalized ED symptoms over a period of 6–12 months (13, 14). There is some preliminary evidence suggesting the effectiveness of FBT for adolescent BN as well (15, 16). However, one drawback to this treatment is that it requires special training in the treatment of eating disorders. While the approach is manualized in a textbook, which is helpful for training, it nonetheless is a 6–12 month intensive treatment best offered by therapists who specialize in the treatment of adolescent eating disorders. Thus, while early intervention is strongly recommended for adolescents who present with mild or subsyndromal EDs, the recommended treatment for EDs may be overly expensive or difficult to access for this population, who may be more likely to present to a nonspecialized community mental health service than to a specialized ED program.

Despite the need for effective treatments and guidelines for treating youth who present in the early stages of an eating disorder, a literature review on early intervention for adolescent EDs or on the use of FBT and community-based approaches for the treatment of mild or subsyndromal adolescent EDs revealed few studies. Two studies examined the use of an online, 6-week, parent-based intervention for adolescents at risk of developing anorexia nervosa (17, 18). Jones et al. (17) examined the feasibility and acceptability of an online program in families of 46 adolescent females either at risk of developing AN or with an ED of duration less than 6-months. The program received positive feedback from parents, and 16 of 19 participants “remained stable or increased in ideal body weight and reported decreased eating disorder attitudes and behaviors” (17). Jacobi et al. (18) used this same intervention to compare patients randomized to either the internet-based intervention *versus* a wait-list control in 66 families of adolescents at high-risk of developing AN (18). Unfortunately, high dropout rates limited the findings of this study. Of the 27 families who completed 12-month follow-up measures, weight gain was significantly higher in the intervention group, although risk profile was no different between groups. Another study was found which examined the use of modified FBT in a primary care setting (19). This study described the treatment of 15 adolescents with low-weight and/or restrictive eating disorders with a mean weight loss of 11.6 kg. Treatment providers included two pediatricians and one nurse practitioner who had received a 4-hour training session based on Phase 1 FBT principles followed by 1-hour monthly consultation sessions. Modifications to the FBT model to allow for delivery within a primary care setting included shortening sessions to 30–45 min, eliminating the family meal and minimizing sibling involvement. Patients received a mean of 9.2 sessions over 3 months. At the end of the study patients had gained an average of 6.76 kg and demonstrated a significant increase in their BMI percentile (19). Thus, these and other strategies for early treatment of EDs are actively being studied, yet to date there remains no evidence-based treatment guidelines for early intervention in adolescent EDs.

FBT is traditionally provided over the course of 20 sessions which take place over approximately 12 months. However, some studies have suggested that reducing the number of sessions or time period over which treatment is provided may be possible (20–23). Lock et al. examined the outcomes for patients who were provided FBT over 10 sessions *vs* the traditional 20 sessions. Outcomes at the end of 12 months and at 4-yr follow-up showed no differences between groups, although it appeared as though those patients with eating-related obsessive compulsive thinking or from non-intact families may benefit more from longer treatment (20, 21). Rockwell et al. (22) described 19 cases of adolescent patients with AN treated in a 5-day intensive FBT treatment program. At follow-up (mean 278.4 days posttreatment), weight had risen from 84 to 99% of goal weight, and all but one patient had experienced sustained weight gain (22). Marzola et al. (23) used a similar protocol of a 5-day intensive therapy for adolescents with AN and restrictive eating disorders. They compared groups in which this therapy was provided to families individually versus in a multi-family format to 74 adolescent patients with AN. At 30-month follow-up, 60.8% of patients were found to be in full remission and an additional 27% were in partial remission. There were no differences noted between the group that received the treatment as single families *vs* multifamily formats (23).

Given the successful treatment of patients with moderate to severe EDs using FBT (9, 12–15), and given the known benefit of early detection and treatment of EDs (7, 8), it is plausible that a short course of modified FBT has an unexplored therapeutic use as an effective treatment for mild or ‘subsyndromal' EDs. Thus, the aim of this study was to retrospectively examine the potential effectiveness of a short targeted intervention, consisting of five modified FBT sessions, designed to treat adolescents with mild EDs before the disease progresses and worsens.

We are based in a medium-sized Canadian city (population 990,000) where specialized care for nonsevere adolescent EDs is largely unavailable within the community. Hospitals such as ours, with multidisciplinary teams specialized in the treatment of pediatric EDs, cannot accommodate the number of referrals for adolescents presenting with EDs, and as such we can only accept patients with severe EDs. Patients with mild or moderate EDs are unfortunately left to seek treatment in the community, where counselors typically lack training or confidence in treating EDs. As a result of this gap in services, an intervention targeted towards adolescents with milder EDs was developed in 2016 by first author W.S. (a child and adolescent psychiatrist). Five innovative modified FBT sessions (termed ‘DREAMS' therapy sessions) were developed based on her 17 years of clinical experience as a noncertified FBT therapist treating youth with severe EDs. Inspired by FBT, the family sessions were developed to address issues of nutrition and ED symptoms, cognitions and urges, as well as mood, anxiety, sleep, school issues, and peer and family relationships. The acronym DREAMS was created based on the themes of the various sessions. In order of the letters of the acronym, but **not** the relevant order of sessions, the themes are **D**epression (mood, self-harm and suicidality), **R**elationships (with family and with peers), **E**ducation (school issues/stressors), **A**nxiety/stress, **M**eals/nutrition/ED symptoms, and **S**leep (and **S**ubstance use if relevant), spelling DREAMS as a memory aid for community counselors.

The objective of this case series was to review the hospital charts of the seven patients with mild EDs who were treated in 2016 by W.S. using the five DREAMS sessions that she had developed. Her progress notes were reviewed by a research student to determine the potential of the DREAMS sessions as an early intervention for youth with mild EDs and to determine whether there is value to further studying this approach. It should be noted that only seven patients were treated because no research funding or extra resources were provided, and as such after treating those seven patients, the team and W.S. had to return to only accepting referrals and providing treatment for patients with severe eating disorders.

## Materials and Methods

### Participants

Participants consisted of seven patients with EDs deemed to be mild in severity who were referred to our hospital-based specialized multidisciplinary pediatric ED program. Under normal circumstances, these referrals would have been denied as their conditions (as described on the referral form) would not have been deemed severe enough for treatment by our team. Instead of being declined, these seven families were offered treatment with the five DREAMS sessions. The progress notes from the 2016 DREAMS sessions of the seven patients included in this case series were examined retrospectively in 2019. This retrospective chart review was approved by our hospital Research Ethics Board.

### Study Protocol

Patients underwent treatment between January and December 2016 in the form of five DREAMS family therapy sessions. The patients and families were not ‘handpicked' but rather consisted of the first seven mild–moderate ED referrals received by the team at the time, which normally would have been rejected and returned to the family physician. The patients who were offered the DREAMS sessions were deemed to be eligible for treatment if they were between 13 and 18 years of age, and had a nonsevere ED that met the following criteria: 1) they were at least 89% of their premorbid body weight; 2) symptoms of bingeing and purging occurred no more than seven times per week; and 3) the patient was taking at least 50% of their daily nutritional needs (OR, if there was more significant restricting, it had only been occurring for less than two months).

All seven families were sent a letter offering them five family therapy sessions, with no further follow-up, and all seven gave written consent for treatment. The patients did not receive a formal assessment by our hospital-based ED team, or by W.S., in order to determine whether a community counselor could feasibly offer these sessions without the need for a psychiatric or psychological assessment (in Canada, these resources can be very difficult for patients to access). A letter also went to the family doctors of the patients informing them that these therapy sessions would be offered to the patient, but that the family physician was responsible for their medical care and follow-up. The DREAMS therapy sessions developed by W.S. can be best described as a form of brief modified FBT, which entailed five sessions across 6 weeks for each patient and their family, with one week between each session, but with a two week break between sessions 4 and 5. The DREAMS sessions were conducted by first author W.S. based on her clinical experience of the most important components of FBT for treating youth with EDs, who often present with comorbid anxiety and depression. The breakdown of each session is as follows:

**Session 1**: (This session represents the ‘**M**' in ‘DREAMS,' as the focus of the session is on meals, nutrition and ED symptoms.) The highlights of session 1 include:

- The therapist raises anxiety by stressing the seriousness of the ED, lifts blame from the patient and parents, externalizes the illness, and empowers parents to contain symptoms of the ED.
- Psychoeducation on EDs is provided to the family, including the importance of nutrition to the well-being of teenagers and the side effects of insufficient nutrition.
- The ED is compared to an ‘OCD-like' illness in that the patient experiences obsessive ED thoughts and then feels compelled to have symptoms to decrease the intensity of the thoughts (and insufficient nutrition exacerbates all of this).
- Families are helped to see the ED as related to stress or to feeling “not good enough.” Patients are helped to describe the stressors or triggers that might be associated with the development of the ED in order to increase empathy in the family.
- Parents are empowered to take charge of nutrition, contain ED symptoms, and supervise meals, in a firm but compassionate manner. If siblings are present, they are encouraged to offer support to the patient. For underweight patients, weight is recorded, and a weight graph is started.
- The family is given a ‘folder' of resources to take home with them, that includes the name of a book for parents (“Help Your Teenager Beat an Eating Disorder”) (24) and a sample 2400 calorie ‘meal plan' (*i.e.* a list of suggestions for snacks and meals, to give parents an idea of what a typical day's intake might look like).

**Session 2**: In keeping with FBT principals, the focus of this session remains on nutritional intake and ED symptoms, in order to stress the key importance of normalizing nutrition, eating, and weight for the patient's recovery. The following topics are covered in session 2:

- The family is asked whether nutrition has improved or if symptoms have decreased, and the patient is weighed, and the weight is graphed if indicated (*i.e.* if there is a history of weight loss, and weight at referral was below premorbid weight.)
- The family is asked about what the patient is eating, eating patterns, meal and post meal supervision times, and any symptoms of bingeing, purging, or exercising.
- The family's efforts to help the patient normalize his or her eating are reviewed, including asking about what is and is not working, praising them for their efforts, and helping them to focus on containing any ongoing ED symptoms.
- The patient is helped to voice their thoughts and feelings associated with the family's efforts to contain the ED symptoms. The therapist works to help separate the patient from the illness, and the conversation remains focused on ED thoughts, urges, and symptoms.

Following this, the family and patient are told that EDs tend to be associated with stress or feeling ‘not good enough,' and they are informed that the brain needs to be well nourished in order to cope with and recover from anxiety, depression, or stress. They are told that now that nutrition has begun to improve (thanks to their efforts), these topics will be covered next week.

**Session 3**: This session (which includes the letters **D**, **A** and **S** from the acronym DREAMS) starts by ensuring that eating is normalizing and ED symptoms are being contained. Lots of praise is given for any improvements. The weight is graphed if indicated (*i.e.* if weight at referral was below premorbid weight, and one goal of treatment is weight gain. Note that patients had to be at least 89% of premorbid weight to be in this study). The therapist then asks the patient about any low mood, worries, or sleep problems. Psychoeducation is provided on the relationship between nutrition, sleep, mood, and anxiety (*e.g.* how insufficient or irregular nutrition can worsen these problems, and these problems in turn can make the ED worse):

- The family is helped to understand that negative feelings ‘fuel' the ED so that as well as containment of symptoms and a focus on improving nutrition, the family can help by supporting the patient to get treatment for anxiety, depression or insomnia and by encouraging self-care. Treatment options are discussed, and the therapist can recommend that the patient consult a physician to consider whether medication might be indicated.
- The family is asked what might be causing the patient to feel sad, worried, angry, or ‘not good enough,' and to problem-solve ideas on how to decrease the negative emotions fueling the ED.
- The patient is encouraged to talk openly about any symptoms of self-harm or suicidality. The counselor helps the family to empathize, express concern, and support, and problem-solve how to keep the patient ‘safe' from these symptoms.

**Session 4**: The patient is weighed again if indicated, and the weight is graphed (if patient is not yet at premorbid weight). A brief discussion can take place regarding weight, intake, and ED symptoms if needed. (Note that unlike FBT for moderate–severe EDs, in which the focus needs to remain on weight gain and containment of ED symptoms, after 3 weeks of symptom containment and improved intake, many patients with mild EDs and a short history of restricted intake will be weight-restored.) Therefore, the main areas of discussion in session 4 are school (educational) issues and relationships (the **E** and **R** in DREAMS). This includes:

- Asking the patient about any stressors associated with school (*e.g.* any social anxiety or anxiety about tests, assignments, class presentations, peer rejection, bullying, *etc*). The therapist explains that while poor nutrition can cause anxiety, anxiety and stress can also fuel the ED symptoms. The family is encouraged to problem solve (*e.g*. by helping to make a homework schedule, reaching out to the teacher or school guidance counselor, using resource rooms, *etc*).
- Ensuring family awareness of any other stressors affecting the patient. The family is again helped to understand that negative feelings ‘fuel' the ED, and that they can help by supporting the patient to feel ‘good enough,' by decreasing stressors, or by improving relationships with peers or at home (which may require tackling conflict in the family).

At the end of the session, the counselor points out any improvements observed, expresses confidence in the patient and family's ability to overcome challenges, and notes that the next session will be the last one. A return session is scheduled in two weeks rather than the usual one week return.

**Session 5**: The following topics are covered in the final session:

- A full and final review of nutrition and ED symptoms is undertaken, and weight graph is reviewed if indicated. The ED is externalized, and if there have been improvements, the patient and family are commended for their role in helping to protect the patient from the devastating effects of an ED.
- Ongoing challenges are discussed (*e.g.* “still afraid to take desserts”), and the patient and family are encouraged to keep the momentum and progress going until eating gets back to “normal.”
- Mood, anxiety, and stressors are reviewed, empathy is provided and modeled for any ongoing challenges or stressors, and praise is given to the family for any improvements and any support the family has provided which the patient has accepted.
- The patient and family are asked to problem-solve what might be some helpful next steps or follow-up now that therapy is ending. For example, patients with ongoing mental health problems might be referred for individual CBT, or a DBT group, or be given information about workbooks or online resources. They could decide it would be helpful to ask the family doctor to monitor weight or to prescribe medication. (If the ED has worsened, the patient should be referred to a specialized ED therapist or team.)
- The counselor ends the session by praising all of them for any improvements made, for their ability to work together, and for their ability to problem-solve. The follow-up plan is carefully described, and the family is tasked with supporting each other and problem-solving any unresolved or new challenges.

### Hypothesis

These five DREAMS therapy sessions were created as a treatment that could be offered to youth who present with mild EDs. The hope was to provide a treatment that, if deemed helpful by these 7 families, could then eventually be studied for its potential effectiveness in treating mild EDs and for its potential utility in training nonspecialized community counselors on how to approach the treatment of youth who present with mild EDs. It was hoped that patients who received the treatment would demonstrate improved mood, anxiety, and sleep, decreased ED symptoms, and for underweight patients, improved weight gain. It was hoped that parents would develop a better understanding and knowledge of EDs and enhanced feelings of efficacy in supporting their child's recovery. Unfortunately, this was not a funded study, and no measures were used. Seven patients were treated by W.S. with the DREAMS family sessions, and their charts were reviewed (by ZA) in an attempt to discern whether or not there is evidence that this treatment is worthy of further study.

## Results

Seven out of eight patients referred for the DREAMS sessions met the inclusion criteria and received the five therapy sessions (the 8^th^ patient was deemed to have a more severe ED and was referred back to the hospital ED team for treatment). The average age of the patients was 15.0 years, with a range from 13 to 17 years at the first DREAMS session. Six of the patients were female and one was male. None of the patients were given a psychiatric assessment or formal psychiatric diagnosis (in keeping with the idea that community counselors should be able to offer this treatment). Three of the patients had symptoms of restricting, bingeing and purging, two had symptoms of restricting and bingeing, one had symptoms of bingeing and purging, and one had symptoms of restricting only (and was 93% of premorbid weight). Along with the ED, all seven patients reported depressed mood (four with self-harm, four with suicidal ideation), and all seven patients described symptoms of an anxiety disorder (six with symptoms of social anxiety, six with generalized anxiety, one with panic attacks). At the first DREAMS session, when asked about the main problem that brought them to treatment, five of the seven patients identified that ED was their main problem that they would like help with, and they expressed motivation to recover (in contrast to many patients with severe EDs, who may not be motivated to recover). Four of the seven parents expressed during the first session that they did not know how to help their child recover from the ED. Six of the seven patients completed all five sessions in an average of 6.33 weeks. (One patient did not complete session 5 as the family felt she had improved enough that they did not need the final session.) Six of the seven families had the patient, mother, and father attend all sessions (for one patient the father missed one session), and one patient also had their brother attend three of the sessions.

Of the seven patients, all reported significant improvements in their ED by the end of the five sessions, with four of the patients being described as in full remission from their ED and with eating described as “completely normal.” One patient was deemed to be 93% of her healthy weight in the first session and had her weight graphed during the sessions; she gained 3.4 kg in 4 weeks and was 98.5% of her goal weight in the final session. Six of the seven patients described improvements to their mood, with two declaring that their depression had resolved. Three of the seven patients described improvement in anxiety, but the remaining four patients stated that their anxiety persisted in the final session. (See **Table 1** Summary of improvements/results).

**Table 1:** Summary of improvements/results documented in the progress notes for each patient in the final session.

	Presenting Symptoms	Physician Notes from Final Session	Family Feedback in Notes from Final Session
**Patient 1**	Restricting, dieting, bingeing, purging, fear of weight gain/wanting to lose weight.	ED and major depressive episode in remission. Anxiety remains severe but coping strategies discussed (*i.e.* deep breathing, relaxation, counseling, yoga). Able to enjoy activities previously could not. Sleep an ongoing problem even with trazodone. Relationships with friends and family good.	“Eating disorder and eating are no longer concerns.” Parents noted improved coping with school anxiety. Overall positive feedback but acknowledgment that anxiety remains a problem. Eating normal, weight normal. Patient reports some ED thoughts but “I push them to the back of my mind.”
**Patient 2**	Restricts and avoids all ‘unhealthy' food but still eating three meals a day, fear of weight gain, wanting to lose weight. 93% of goal weight. Strong ED thoughts and urges. Also not attending school due to severe anxiety	Now 98.5% of goal weight. Improved coping with anxiety, improved self-esteem, denies any eating disorder thoughts/urges now. Eating, mood, sleep and friends all going well. Still has severe social anxiety and school avoidance.	Huge improvements in eating; no longer restricts ‘unhealthy' foods, eating is ‘completely normal.” “Seems much happier these days.” Noted improvements in “maturity.” Family relationships reported as positive and family was thankful for the sessions.
**Patient 3**	Restricting, bingeing, fear of weight gain, exercising to lose weight, body image concerns, recent self-harming.	Eating improved tremendously; eating now described as ‘normal'. No longer exercising to lose weight, no longer bingeing. Increased anxiety and low mood associated with school starting. No more suicidal ideation or self-harm. Patient gaining ability to voice concerns and has increased coping strategies.	Parents felt empowered to take charge of nutrition and make it mandatory, and patient reported this as helpful. Eating now normalized. Patient reported the ED thoughts and urges are there but decreased from before. Relationship with friends and family remain supportive.
**Patient 4**	Restricting/skipping meals, bingeing, recent suicide attempt.	Full remission of eating disorder; denies any ED thoughts whereas these previously took up 80% of brain. Patient eats well and normally, with no avoidance of foods. No trouble sleeping, mood is good whereas previously very low with suicidal ideation and self-harm. Denies significant anxiety whereas previous severe social anxiety.	Very positive feedback from family due to increased mood and full remission of ED symptoms.
**Patient 5**	Restricting, avoiding unhealthy food, bingeing, purging, fear of weight gain, exercising to lose weight, body image concerns.	Nutrition was improved and weight approaching healthy weight. Patient learned to eat challenging food like cake for dessert. Significant body image concerns and ED thoughts and urges still present but now copes by trying to push them away and distract.	Patient reports “mood was improved, anxiety had decreased, confidence had improved, was being more independent and more social with friends.” Patient and both parents describe significant improvement in all areas, including mood, anxiety and ED.
**Patient 6**	Bingeing (7×/week), purging (7×/week), fear of weight gain, exercising to lose weight, body image concerns, history of self-harm and suicidality.	Mood 3/10 midtreatment, now improved to 6–7/10, with no self-harm or suicidality. Bingeing and purging decreased to 3×/week. Expresses more positive outlook, now motivated to recover from ED.	Patient slightly more irritable from feeling parents are being overly controlling. Overall thankful for sessions and hopeful for recovery.
**Patient 7**	Restricting, bingeing, purging, body image concerns, history of self-harm and suicidal ideation and previous suicide attempt.	Decreased anxiety. ED thoughts and urges still there but no symptoms, *i.e.* no restricting, bingeing, or purging. Eating is normalized, not avoiding any foods; at healthy weight. Mood is good, no self-harm or suicidality. Enjoys spending time with friends. Relationships at home and with friends are very good.	Eats three meals and one snack per day but parents feel patient's eating is “not yet 100%” because she will not choose to eat and sometimes resists. Parents felt patient had made significant progress and parents had learned to help her. Improved family communications and support.

Some key factors contributing to the success of the DREAMS sessions that were identified by the families included: parents providing consistent praise and support, parents taking control of meals and making nutrition “mandatory,” finding creative solutions to problems (*e.g.* having the patient create videos to show parents that they had eaten when parents could not be home), and coping strategies that were discussed in the sessions. Some challenges to recovery identified by patients and families included a patient feeling as if parents were ‘lecturing' them as opposed to supporting them, and some adolescents feeling as if parents were being overly controlling and telling them what to do.

## Discussion

Despite strong evidence for the importance of early intervention in the treatment of adolescent EDs (8), there are no guidelines or recommended treatments for youth with new onset, subsyndromal or mild eating disorders and little is known about what factors affect outcomes in this population. According to Currin and Schmidt, “Whilst existing research provides preliminary indication that early intervention for EDs may be useful, not enough is known about the variables critical for ensuring good outcomes” (25).

In addition, despite the evidence for the importance of early intervention, there are also many communities, including our own, where access to treatment for EDs is limited to specialized hospital-based programs that only treat those with moderately severe to severe eating disorders. (In Ontario, Canada, these specialized programs are evidence-based and offer FBT to most children and adolescents who present with restrictive EDs.) Thus, many young people with new onset or mild EDs receive no treatment at all until the ED has progressed. Others seek treatment in the community, where many nonspecialized community mental health counselors lack experience, training, or confidence in treating EDs.

The five DREAMS sessions were developed in an attempt to address this problem. They were based on the first author's experience of using FBT to treat severe EDs in adolescents and were created using the ‘principles' of FBT. They were designed to be replicable and consistent such that they could potentially be taught to nonspecialized mental health counselors as a short-term intervention for adolescents who present with mild or new onset EDs. (It is expected that these patients will also be followed by their family physicians.) The purpose of this case series was to review the progress notes of the seven patients who received the DREAMS sessions, in order to determine whether this treatment is worthy of further study. Based on the results, the treatment does seem to have been effective in improving ED symptoms and mood in these patients. For some but not all, sleep and anxiety also improved.

There are significant limitations to this case series, including the small sample size, lack of reliable and valid measures, lack of a comparison group, possibility of bias, and possibility that the outcomes were positive as a result of the author's experience in treating EDs (and thus perhaps not replicable if treatment were to be provided by an inexperienced counselor). Nonetheless, the positive results based on what was written in the progress notes (including feedback from families) suggest that these five family therapy sessions are worthy of further study as a possible treatment for adolescents with mild EDs. Prior to further research, it would be worth modifying the current sessions based on feedback from other ED experts and from families who have had previous ED treatment, and then documenting in detail the proposed content of the five sessions in order to make them replicable. Funding could then be obtained to more formally study the effectiveness of this intervention in a randomized controlled trial while also studying the effectiveness of the intervention as a training tool for community counselors who lack experience in the treatment of adolescent EDs.

## Conclusion

It is imperative that we find effective, brief, innovative treatments that will prevent youth with mild or new onset EDs from deteriorating and developing more severe or chronic EDs. It is also imperative that we train nonspecialized counselors in the community to recognize and treat these illnesses. Further research is needed into early interventions for adolescent EDs, such as the five FBT-inspired DREAMS sessions described in this paper.

## Data Availability Statement

The data that support the findings of this study are available on request from the corresponding author, WS. Data is not publicly available as it would compromise the privacy of research participants through the release of identifiable information.

## Ethics Statement

This study involving human participants was reviewed and approved by the CHEO Research Institute Research Ethics Board. Written informed consent to participate in this study was provided by the participants and by the participants' legal guardians/next of kin.

## Author Contributions

WS created the DREAMS sessions, treated the patients, designed the study, wrote the protocol, and wrote much of the first draft of the manuscript. ZA wrote the REB proposal, created the data extraction form, extracted the data from the charts, and wrote some of the first draft of the manuscript. MN and LI commented on the protocol. BB created an Excel sheet of the data, summarized the data, and wrote the results section. ZA and BB conducted literature reviews. DV, ZA, and BB created the reference list, ZA and BB conducted literature reviews and LI summarized some of the relevant literature. DV helped with formatting the manuscript. All the authors commented on the manuscript. All the authors contributed to and have approved the final manuscript.

## Funding

This research was funded in part by the CHEO Psychiatry Associates Research Fund, who provided a $5,000 grant to hire a summer student (ZA) to conduct a literature review, submit a REB proposal including any relevant forms, and to extract relevant data from patient charts describing the cases, treatment, and outcomes.

## Conflict of Interest

The authors declare that the research was conducted in the absence of any commercial or financial relationships that could be construed as a potential conflict of interest.
